# DHCR24 Knock-Down Induced Tau Hyperphosphorylation at Thr181, Ser199, Thr231, Ser262, Ser396 Epitopes and Inhibition of Autophagy by Overactivation of GSK3β/mTOR Signaling

**DOI:** 10.3389/fnagi.2021.513605

**Published:** 2021-04-21

**Authors:** Xiaojing Bai, Junfeng Wu, Mengqi Zhang, Yixuan Xu, Lijie Duan, Kai Yao, Jianfeng Zhang, Jimei Bo, Yongfei Zhao, Guoxiong Xu, Hengbing Zu

**Affiliations:** ^1^Department of Neurology, Jinshan Hospital, Fudan University, Shanghai, China; ^2^The Research Center for Clinical Medicine, Jinshan Hospital, Fudan University, Shanghai, China

**Keywords:** DHCR24, hyperphosphorylation, autophagy, PI3-K, GSK3β, mTOR, Alzheimer's disease 3/, Cholesterol

## Abstract

Accumulating evidences supported that knock-down of DHCR24 is linked to the pathological risk factors of AD, suggesting a potential role of DHCR24 in AD pathogenesis. However, the molecular mechanism link between DHCR24 and tauopathy remains unknown. Here, in order to elucidate the relationship between DHCR24 and tauopathy, we will focus on the effect of DHCR24 on the tau hyperphosphorylation at some toxic sites. In present study, we found that DHCR24 knock-down significantly lead to the hyperphosphorylation of tau sites at Thr181, Ser199, Thr231, Ser262, Ser396. Moreover, DHCR24 knock-down also increase the accumulation of p62 protein, simultaneously decreased the ratio of LC3-II/LC3-I and the number of autophagosome compared to the control groups, suggesting the inhibition of autophagy activity. In contrast, DHCR24 knock-in obviously abolished the effect of DHCR24 knock-down on tau hyperphosphrylation and autophagy. In addition, to elucidate the association between DHCR24 and tauopathy, we further showed that the level of plasma membrane cholesterol, lipid raft-anchored protein caveolin-1, and concomitantly total I class PI3-K (p110α), phospho-Akt (Thr308 and Ser473) were significantly decreased, resulting in the disruption of lipid raft/caveola and inhibition of PI3-K/Akt signaling in silencing DHCR24 SH-SY5Y cells compared to control groups. At the same time, DHCR24 knock-down simultaneously decreased the level of phosphorylated GSK3β at Ser9 (inactive form) and increased the level of phosphorylated mTOR at Ser2448 (active form), leading to overactivation of GSK3β and mTOR signaling. On the contrary, DHCR24 knock-in largely increased the level of membrane cholesterol and caveolin-1, suggesting the enhancement of lipid raft/caveola. And synchronously DHCR24 knock-in also abolished the effect of DHCR24 knock-down on the inhibition of PI3-K/Akt signaling as well as the overactivation of GSK3β and mTOR signaling. Collectively, our data strongly supported DHCR24 knock-down lead to tau hyperphosphorylation and the inhibition of autophagy by a lipid raft-dependent PI3-K/Akt-mediated GSK3β and mTOR signaling. Taking together, our results firstly demonstrated that the decrease of plasma membrane cholesterol mediated by DHCR24 deficiency might contribute to the tauopathy in AD and other tauopathies.

## Introduction

Previous studies found that there is a significant reduction in the expression of new gene in specific vulnerable brain regions in Alzheimer's disease (AD) patients, which was named selective AD indicator 1 (Seladin-1), or 3β-hydroxysterol-Δ24 reductase (DHCR24) (Greeve et al., 2000; Waterham et al., 2001). In the past more than 10 years, increasing data suggested that the downregulation of DHCR24 expression could was induced by many AD-related risk factors, including genetic, β-amyloid protein (Aβ), aging, intermittent low or high blood glucose, hyperglycemia, hyperinsulinemia, diabetes mellitus, chronic inflammation, oxidative stress, obesity, metoblic syndrome, and growth factor insufficiency (e.g., insulin, IGF-1), etc. (Greeve et al., 2000; Livonen et al., 2002; Giannini et al., 2008; Kuehnle et al., 2008; McGrath et al., 2009; Khuda et al., 2010; Vanmierlo et al., 2010; Berisha et al., 2011; Shih et al., 2011; Sharpe et al., 2012; Ing et al., 2014; Kleinridders et al., 2014; Orre et al., 2014; Sassi et al., 2014; Bing et al., 2015; Kazkayasi et al., 2016; Najem et al., 2016; Dhana et al., 2018; Lemche, 2018). Accumulating evidences supported that the downregulation of DHCR24 is linked to the pathological risk factors of both sporadic and familiar AD, suggesting a potential role of DHCR24 in AD pathogenesis. Moreover, studies indicate that DHCR24 transcription is selectively down-regulated in brain regions vulnerable to Alzheimer's disease in the transgenic mouse model of AD (Livonen et al., 2002; Vanmierlo et al., 2010; Orre et al., 2014). Besides, in AD patients, it has been reported that DHCR24 transcription and protein expression were selectively down-regulated in the brain areas affected in AD (Greeve et al., 2000; Livonen et al., 2002; Liang et al., 2008). Thus, it seems that DHCR24 is likely to establish a new direct link between the familial and sporadic AD. Nevertheless, the potential role of DHCR24 in Alzheimer disease and other neurodegenerative diseases is still unknown (Greeve et al., 2000; Livonen et al., 2002; Sharpe et al., 2012; Zerenturk et al., 2013). Iivonen et al. found that the downregulation of DHCR24 might be associated with hyperphosphorylated tau in AD mouse model, but the molecular mechanism behind this association remains unknown (Livonen et al., 2002). Tauopathy is regarded as one of the most characterized pathology in AD and other tauopathies, which is recognized as linkage to hyperphosphorylated tau, immunohistochemically detected paired helical filament tau, and neurofibrillary tangles (Alonso and Cohen, 2018). Therefore, to elucidate a molecular mechanism link between DHCR24 and tauopathy will help to clarify the role of DHCR24 in the pathogenesis of AD and other tauopathies.

DHCR24 is known as the key synthetase in cholesterol synthesis, which catalyzes the final step of cholesterol synthesis via the Bloch pathway, or converts lanosterol to cholesterol at the first step in the Kandutsch-Russell pathway (Drzewińska et al., 2009; Segatto et al., 2019). DHCR24 mutation results in Deficient/defective of DHCR24 activity, low cholesterol, high desmosterol levels, and disturbance of cholesterol-rich lipid-rafts in brain (Waterham et al., 2001; Zerenturk et al., 2013). Moreover, recent studies suggested that activities of DHCR24 enzyme can obviously affect membrane lipid raft organization and dynamics, which are key factors for signaling processes for cellular responses (Crameri et al., 2006; Lu et al., 2006; Matthews et al., 2008; Gao et al., 2011). Therefore, the present investigation is to explore *whether the* downregulation of DHCR24 may induce an abnormality of membrane lipid raft-dependent PI3-K/Akt signaling, including downstream glycogen synthase kinase-3β (GSK3β) and mammalian target of rapamycin (mTOR) signaling, which are involved in the abnormal hyperphosphorylation of some tau sites in tauopathy. In our experiment, we will focus on the effect of DHCR24 on the tau hyperphosphorylation at some sites such as Thr181, Ser199, Thr231, Ser262, Ser256, Ser396, and autophagy, in order to elucidate the relationship between DHCR24 downregulation and tauopathy.

## Materials and Methods

Primary antibodies against DHCR24 (#2033), AKt (#4691), Phospho-Akt (T308) (#13038), Phospho-Akt (Ser473) (#4060), PI3 Kinase p110α (#4249), GSK-3β (#12456), Phospho-GSK-3β(Ser9) (#5558), mTOR (#2983), Phospho-mTOR (#2971), Tau (#46687), Phospho-Tau (Thr181) (#12885), Phospho-Tau (Ser199) (#29957), Phospho-Tau (Ser396) (#9632), LC3B (#3868), P62/SQSTM1 (#23214), GAPDH (#2118), secondary antibodies anti-rabbit IgG, HRP-linked antibody (#7074), Anti-rabbit IgG (H+L) (#5366), Anti-mouse IgG, HRP-linked antibody (#7076) were purchased from Cell Signaling Tech (USA), Primary antibodies against Caveolin-1 (ab2910), Phospho-Tau (Ser262) (ab131354) were purchased from British abcam Company, Primary antibodies against Phospho-Tau (Thr231) (4137) was purchased from China ABclonal Company. Primers were purchased from Sangon Biotech (Sangon Biotech, China). Propidium Iodide (abs9105), Methyl-β-cyclodextrin (abs42021762), Filipin III (abs42018484) were purchased from China Absin Company.

### Cell Culture

SH-SY5Y cells were obtained from Chinese Academy of Sciences (Shanghai, China) and kept in DMEM F12 (Corning, USA) with 10% fetal bovine serum (Gibco, USA). Following ATCC protocols, all cells were cultured in a 5% CO2 humidified incubator at 37°C.

### Lentivirus Transfection and Screening of a Stable SH-SY5Y Cell Line

With regard to lentivirus transfection, lentivirus at multiplicity of infection (MIO) about five mediated DHCR24 shRNA, DHCR24 cDNA, and NC infected into the SH-SY5Y cells. At 48 h after infection, the medium was replaced with fresh complete growth medium containing the appropriate concentration of puromycin (6 μg/mL). At 3 to 4 days after infection, the selected cells were used for experiments. Note: ShRNA target sequence was as follow: (5'-GCATCATCCCTGCCAAGAAGT-3'); The empty vector sequence was as follow: (5'-TTCTCCGAACGTGTCACGT-3'), cDNA sequence was as follow: (5' -ATGGAGCCCGCCGTGTCGCTGGCCGTGTGCGCGCTGCTCTTCCTGCTGTGGGTGCGCCTGAAGGGGCTGGAGTTCGTGCTCATCCACCAGCGCTGGGTGTTCGTGTGCCTCTTCCTCCTGCCGCTCTCGCTTATCTTCGATATCTACTACTACGTGCGCGCCTGGGTGGTGTTCAAGCTCAGCAGCGCTCCGCGCCTGCACGAGCAGCGCGTGCGGGACATCCAGAAGCAGGTGCGGGAATGGAAGGAGCAGGGTAGCAAGACCTTCATGTGCACGGGGCGCCCTGGCTGGCTCACTGTCTCACTACGTGTCGGGAAGTACAAGAAGACACACAAAAACATCATGATCAACCTGATGGACATTCTGGAAGTGGACACCAAGAAACAGATTGTCCGTGTGGAGCCCTTGGTGACCATGGGCCAGGTGACTGCCCTGCTGACCTCCATTGGCTGGACTCTCCCCGTGTTGCCTGAGCTTGATGACCTCACAGTGGGGGGCTTGATCATGGGCACAGGCATCGAGTCATCATCCCACAAGTACGGCCTGTTCCAACACATCTGCACTGCTTACGAGCTGGTCCTGGCTGATGGCAGCTTTGTGCGATGCACTCCGTCCGAAAACTCAGACCTGTTCTATGCCGTACCCTGGTCCTGTGGGACGCTGGGTTTCCTGGTGGCCGCTGAGATCCGCATCATCCCTGCCAAGAAGTACGTCAAGCTGCGTTTCGAGCCAGTGCGGGGC CTGGAGGCTATCTGTGCCAAGTTCACCCACGAGTCCCAGCGGCAGGAGAACCACTTCGTGGAAGGGCTGCTCTACTCCCTGGATGAGGCTGTCATTATGACAGGGGTCATGACAGATGAGGCAGAGCCCAGCAAGCTGAATAGCATTGGCAATTACTACAAGCCGTGGTTCTTTAAGCATGTGGAGAACTATCTGAAGACAAACCGAGAGGGCCTGGAGTACATTCCCTTGAGACACTACTACCACCGCCACACGCGCAGCATCTTCTGGGAGCTCCAGGACATTATCCCCTTTGGCAACAACCCCATCTTCCGCTACCTCTTTGGCTGGATGGTGCCTCCCAAGATCTCCCTCCTGAAGCTGACCCAGGGTGAGACCCTGCGCAAGCTGTACGAGCAGCACCACGTGGTGCAGGACATGCTGGTGCCCATGAAGTGCCTGCAGCAGGCCCTGCACACCTTCCAAAACGACATCCACGTCTACCCCATCTGGCTGTGTCCGTTCATCCTGCCCAGCCAGCCAGGCCTAGTGCACCCCAAAGGAAATGAGGCAGAGCTCTACATCGACATTGGAGCATATGGGGAGCCGCGTGTGAAACACTTTGAAGCCAGGTCCTGCATGAGGCAGCTGGAGAAGTTTGTCCGCAGCGTGCATGGCTTCCAGATGCTGTATGCCGACTGCTACATGAACCGGGAGGAGTTCTGGGAGATGTTTGATGGCTCCTTGTACCACAAGCTGCGAGAGAAGCTGGGTTGCCAGGACGCCTTCCCCGAGGTGTACGACAAGATCTGCAAGGCCGCCAGGCACTGA-3').

### Real-Time PCR Analysis

Total RNA was extracted using RNAiso Plus (Takara, Japan) according to the manufacturer's instructions. Reverse transcription was performed using a PrimeScript™ RT Master Mix (Takara, Japan) with specific primers (see Table 1). Reaction conditions were followed as 37°C 15 min, 85°C 5 s, 4°C 30 min. PCR amplification was performed at 95°C 10 min, followed by 95°C 5 s and 60°C 31 s for 40 cycles using SYBR Green Master. Assays were conducted in triplicate and were repeated at least three times. The amount of DHCR24 normalized to an endogenous control GAPDH given by 2-ΔΔCt, in which threshold cycle (Ct) was obtained using Sequence Detection Software V1.4 (7,300 Real Time PCR System, Applied Biosystems, USA).

**Table 1:** The PCR primer sequences.

**gene**	**Sequence**
DHCR24	
Forward primer	5′-3′ AGTCCAGTTCCCCGTTTA
Reverse primer	5′-3′ CTTACCCAGCACCTTCAA
GAPDH	
Forward primer	5′-3′ ACGGATTTGGTCGTATTGGG
Reverse primer	5′-3′ CGCTCCTGGAAGATGGTGAT

### Immunofluorescence Staining

SH-SY5Y cells seeded in twelve-well-plate were incubated for 12-20 h, followed by washing three times with PBS, then fixed with 4% paraformaldehyde at room temperature for 30 min, followed by washing three times with PBS.0.5% Triton X-100 was adopted to permeate cells for 20 min. Cells were blocked with 5% bovine serum Albumin (BSA) in PBS at room temperature for 1 h, then incubated with the primary antibody at 4°C overnight followed by three times washing with PBS. Samples were incubated in secondary antibody in the dark for 1 h followed by stained with DAPI (0.5 μg/mL) for 5 min at room temperature. Finally, cells were observed and the fluorescent images were captured by a confocal microscope (Leica sp5, Germany).

### Western Blot Analysis

After designated treatment, lysates were generated by placing the cells in SDS lysis buffer containing a cocktail of protease and phosphatase inhibitors. BCA was performed to determine total protein concentrations, which were normalized to 1 mg/mL for all samples. Samples were then prepared in sample buffer and heated to 100°C for 8 min. Equal amounts of lysates were fractionated using 8–12% sodium dodecyl sulfate polyacrylamide gels and were then electrotransferred onto nitrocellulose membranes. Gels were run at a constant voltage (80V) for 2–3 h for maximum separation. Wet transfer was performed for 120 min at constant current (250 mA) using polyvinylidenedifluoride membrane presoaked in methanol. The membrane was blocked in 5% milk in 0.2% TBST. The membrane was then washed three times in TBST for 10 min each. After overnight incubation at 4°C with the primary antibodies [DHCR24 1:1000, Akt 1:1000, Phospho-Akt 1:1000, mTOR 1:1000, Phospho-mTOR 1:1000, LC3B 1:1000, P62/SQSTM1 1:1000, PI3-K 1:1000, GSK3β 1:1000, Phospho-GSK3β 1:1000, Tau 1:1000, Phospho-Tau 1:1000, Caveolin-1 1:500, GAPDH 1:5000] the blots were washed and exposed for 1 h at room temperature to corresponding HRP-conjugated secondary antibodies. Chemiluminescent (Bio-Rad, Hercules, CA, USA) detection was then used to detect the expression of each protein; GAPDH level served as internal loading controls.

### Cholesterol Labeling With Filipin III and Analysis

Cells were grown on coverglass bottom dishes. In order to allow a better visualization of intracellular cholesterol pools, cells were treated with 10 mM methyl-β-cyclodextrin (MβCD, China Absin Company) for 30 min at 37°C to remove cholesterol from the plasma membrane. Cells were then fixed for 10 min at RT with 4% paraformaldehyde in phosphate-buffered saline (PBS). Cells were incubated with a solution of filipin (0.1 mg/ml, China Absin Company) for 30 min. Finally, after two washes, cells were counterstained with propidium iodide (PI, 0.35 μg/ml in PBS; China Absin Company) for 5 min. Acquiring images with a confocal laser scanning microscope equipped with a 380 nm optically pumped semiconductor laser (Leica sp5, Germany). Whole-cell cholesterol staining with filipin did not require MβCD treatment.

### Transmission Electron Microscopy (TEM) Analysis

SH-SY5Y cells were treated by stationary liquid which contained 3% glutaraldehyde and 0.22 mmol/L sucrose phosphate buffer for 4 h, then rinsed three times with 0.1 M phosphoric acid rinsing solution for 10–15 min, and fixed with 1% citric acid for 2 h. Next, stepwise ethanol dehydration, followed by epoxy resin embedding, overnight in a 30°C oven, then in a 60°C oven for 12 h. Using an ultra-thin slicer, 50-to 100-nm-thick slices were cut. Using uranium acetate and lead nitrate double staining, a transmission electron microscopy (Hitachi HT7700, Japan) was used to examine autophagosomes in SH-SY5Y cells.

### Statistical Analysis

Statistical analysis was performed using SPSS statistical software. All data were expressed as mean ± SD from at least three independent experiments. *P*-values were determined using one-way ANOVA. Significance was defined as *P* < 0.05.

## Results

### ShRNA-Mediated Knock-Down and cDNA-Mediated Knock-in of DHCR24 Gene Expression in SH-SY5Y Cells

**Figure 1:**
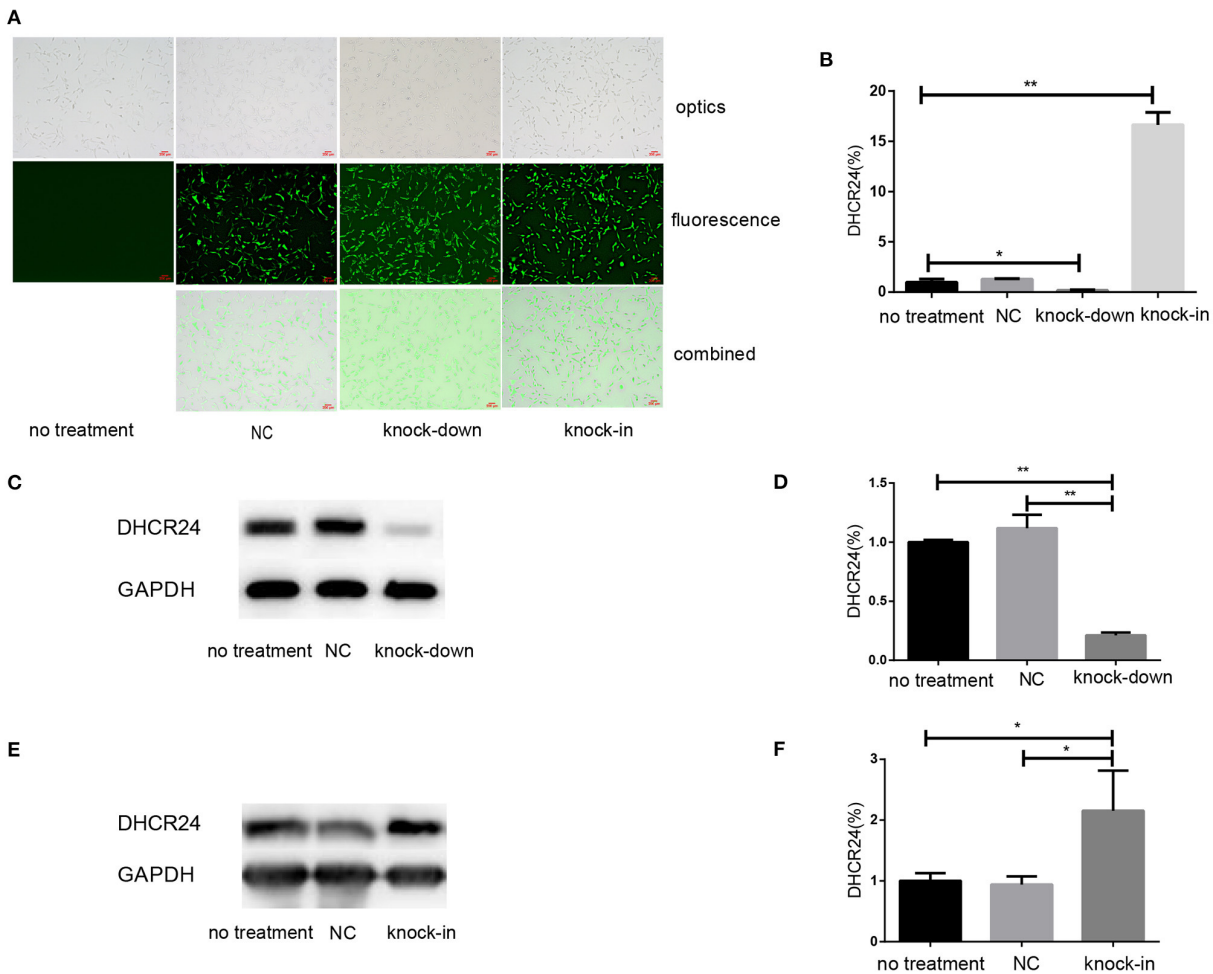
Illustration of SH-SY5Y cells transfected with DHCR24 shRNA-lentivirus and DHCR24 cDNA-lentivirus. The SH-SY5Y cells were transfected with the lentivirus-delivered DHCR24 shRNA, DHCR24 cDNA and lentiviral vector, and used as knock-down group, knock-in group, vector control group (Negative control group, NC) respectively. The untransfected SH-SY5Y cells were used as the no treatment group. **(A)** DHCR24 shRNA, DHCR24 cDNA and lentiviral vector were well-expressed in the SH-SY5Y cells, as indicated by GFP (green) under fluorescence microscopy. Scale bars = 200 μm; **(B)** qPCR analyses were performed and displayed a five-fold lower expression of the DHCR24 shRNA transgenes and a 15-fold higher expression of the DHCR24 cDNA transgenes. Western blot was performed to analyze the knock-down **(C,D)** and knock-in **(E,F)** of DHCR24. All the data are presented as mean ± SD of at least three individual experiments (**B–F**; *n* = 3). *P < 0.01, **P < 0.001; vs. the no treatment and NC group (**C**, Figure 2E, Figures 6A,Q share one loading control).

To explore the influence of DHCR24 on tauopathy in Alzheimer's disease, a transient knock-down of DHCR24 by lentivirus-mediated shRNA (knock-down group) and knock-in of DHCR24 by lentivirus-mediated cDNA (knock-in group) in the SH-SY5Y cell line was performed. DHCR24 shRNA, DHCR24 cDNA were well-expressed in SH-SY5Y cells, as indicated by GFP (green) under fluorescence microscopy (Figure 1A). Transduced cells that survived puromycin selection were followed by qPCR and Western blot analysis. Compared to vector control group (negative control group, NC) and no treatment group (untransfected SH-SY5Y cells), the level of DHCR24 of knock-down group were significantly reduced, and the level of DHCR24 of knock-in group were significantly increased (Figures 1B–F). As mentioned above, the knock-down of DHCR24 by shRNA-lentivirus and knock-in of DHCR24 by cDNA-lentivirus were validated in SH-SY5Y cells.

### DHCR24 Knock-Down Induced Hyperphosphorylation of Tau at Thr181, Ser199, Thr231, Ser262, and Ser396

**Figure 2:**
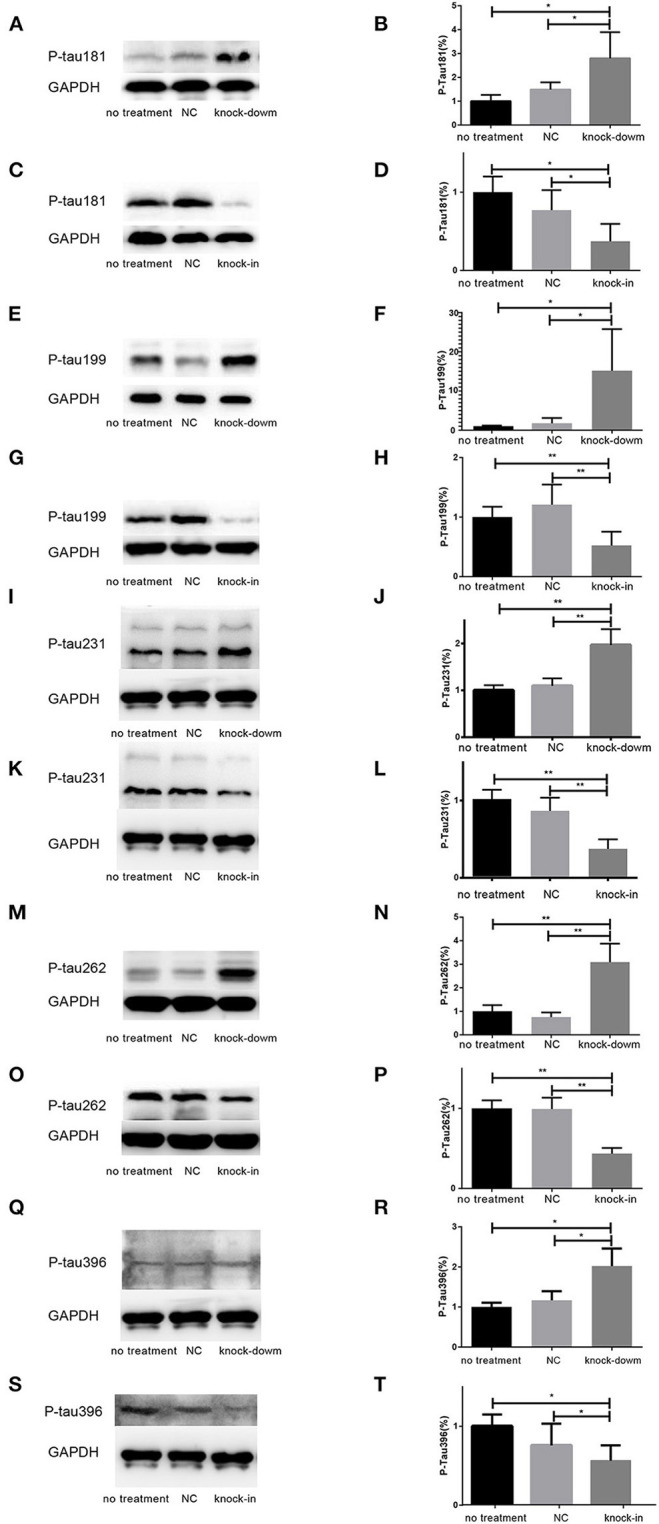
DHCR24 knock-down induced hyperphosphorylation of Tau protein at Thr181, Ser199, Thr231, Ser262, and ser396 in SH-SY5Y cells. **(A,B,E,F,I,J,M,N,Q,R)** Representative western blots of phosphorylated tau at residues Thr181, Ser199, Thr231, Ser262, and Ser396 from DHCR24 knock-down SH-SY5Y cells. **(C,D,G,H,K,L,O,P,S,T)** Representative western blots of phosphorylated tau at residues Thr181, Ser199, Thr231, Ser262, and Ser396 from DHCR24 knock-in SH-SY5Y cells. All the data are presented as mean ± SD of at least three individual experiments (*n* = 3). *P < 0.05, **P < 0.01; vs. the no treatment and NC group (Figures 2I,Q,3E share one loading control).

So far, it is still not to elucidate the potential relationship between DHCR24 and tau hyperphosphorylation. Increasing data supported that the hyperphosphorylation of tau at several sites, such as Thr181, Ser199, Thr231, Ser262, Ser356, and Ser396, which are tightly associated with tauopathy in AD and other tauopathies (Sengupta et al., 1998; Duka et al., 2013; Neddens et al., 2018). Thus, we examined whether the DHCR24 knock-down could induce the tau hyperphosphorylation at some toxic sites, such as Thr181, Ser199, Thr231, Ser262, Ser356, and Ser396. In present study, total tau and phosphorylated tau (p-tau) at residues Thr181, Ser199, Thr231, Ser262, Ser356, and Ser396 were assessed by western blot, respectively. Compared with the no treatment or negative control group, we found that the level of four phosphorylated tau, including tau sites at Thr181, Ser199, Thr231, Ser262, and Ser396, are markedly increased by DHCR24 knock-down in SH-SY5Y cells (Figures 2A,B,E,F,I,J,M,N,Q,R). Moreover, the increase in phosphorylation relative to controls was highest at Thr231 and Ser262. Conversely, after DHCR24 knock-in in human neuroblastoma SH-SY5Y cells, we showed that the level of the above five phosphorylated tau are obviously lowered compared to the control group (Figures 2C,D,G,H,K,L,O,P,S,T). By contrast, the level of p-tau at Ser356 showed no significant difference in four different groups (data not shown). To sum up, we firstly demonstrated that DHCR24 knock-down induced the abnormal hyperphosphorylation of tau at Thr181, Ser199, Thr231, Ser262, and Ser396.

### DHCR24 Knock-Down Obviously Induced the Inhibition of Autophagy

**Figure 3:**
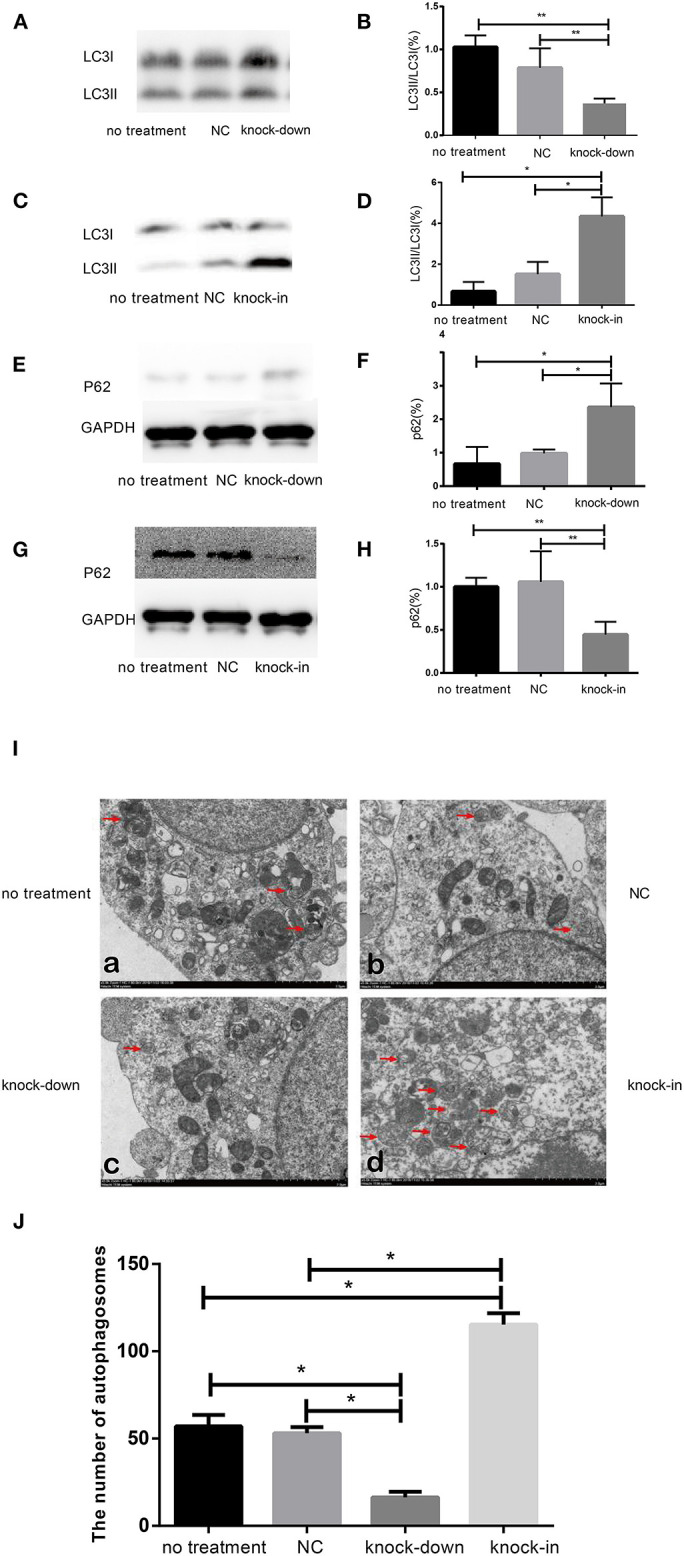
DHCR24 knock-down obviously induced the inhibition of autophagy in SH-SY5Y cells. **(A,B,E,F)** Representative western blots of LC3 and p62 from DHCR24 knock-down SH-SY5Y cells. **(C,D,G,H)** Representative western blots of LC3 and p62 from DHCR24 knock-in SH-SY5Y cells. **(I)** Transmission electron microscopy showed the autophagosomes (red arrow). Scale bar = 2.0 μm. **(J)** Quantitation analyses of autophagosomes for **(I)**. All the data are presented as mean ± SD of at least three individual experiments (*n* = 3). *P < 0.05, **P < 0.01; vs. the no treatment and NC group (Figures 2I,Q,3E share one loading control; Figures 2K,S,3G share one loading control).

To further uncover the role of autophagy in the tauopathy induced by DHCR24 knock-down, we examined the regulation of autophagic activity in the knock-down or knock-in of DHCR24 by transmission electron microscope (TEM) and western blot analysis. Autophagy is an intracellular degradation pathway essential for cellular and energy homoeostasis, involving in the clearance of misfolded proteins and damaged organelles (Lee et al., 2013; Zare-Shahabadi et al., 2015). Nevertheless, with the tauopathy progression, autophagy mediates tau clearance and homeostasis mechanism. Autophagy deficits are regarded as major contributors to the pathogenesis of tauopathy (Yang and Klionsky, 2010; Lee et al., 2013; Zare-Shahabadi et al., 2015). Furthemore, p62, also well-known as sequestosome 1 (SQSTM1) in humans, is a scaffold protein. It interacts with phagophores through the LC3-interacting domain and with the ubiquitinated protein aggregates through the ubiquitin-associated domain. It sequesters the target cargo into inclusion bodies by its PB1 domain. P62 can directly interact with LC3 for autophagosome formation (Yang and Klionsky, 2010; Zare-Shahabadi et al., 2015; Zhang et al., 2017). Thus, p62 and LC3-I/LC3-II are key markers of autophagy. In experiments, as shown in Figures 3E,F, compared with the control groups, we found that the accumulation of p62 was rapidly increased by DHCR24 knock-down in SH-SY5Y cells, suggesting the weakness of autophagy activity. In contrast, the p62 protein level was significantly decreased in the DHCR24 knock-in SH-SY5Y cells, leading to the enhancement of autophagy activity compared to the control groups (Figures 3G,H). Moreover, the ratio of LC3-II/LC3-I in DHCR24 knock-down SH-SY5Y cells was significantly decreased, suggesting the weakness of autophagy (Figures 3A,B). On the contrary, the DHCR24 knock-in obviously increased the ratio of LC3-II/LC3-I, resulting in the enhancement of autophagy in SH-SY5Y cells (Figures 3C,D). Collectively, our data strongly suggested that the DHCR24 knock-down lead to the inhibition of autophagic activity in SH-SY5Y cells.

In addition, to further study the change f autophagosomes, SH-SY5Y neuroblastoma cells were analyzed by transmission electron microscopy. As shown in Figures 3I,J typical autophagosomes of SH-SY5Y cells were observed by transmission electron microscope in control group, knock-down and knock-in groups. It can be seen from the TEM images of autophagosome that most of the autophagosome had the characteristics of double layer or multilayer membrane and inclusions, which could be identified from the other cell structure (shown in Figure 3I). Moreover, double layer or multilayer membrane and inclusions are the characteristics of the autophagosome structure which contains organelles and other cytoplasm components. Additionally, the qualitative analysis of autophagosome was made based on the data from transmission electron microscope. We found that DHCR24 knock-down decreased autophagosome numbers in DHCR24 knock-down SH-SY5Y cells compared to the control groups. On the contrary, DHCR24 knock-in increased autophagosome numbers compared to the control groups. However, TEM analysis revealed that there was no statistically significant difference in the number of autophagosome between the no treatment and negative control group (Figure 3I). Consequently, the results of TEM analysis also further revealed DHCR24 knock-down markedly decreased the formation and accumulation of autophagosome (Figure 3J), suggesting the inhibition of autophagic activity in SH-SY5Y cells. Taking together, our findings strongly supported that the DHCR24 knock-down lead to the inhibition of autophagy.

### DHCR24 Knock-Down Lead to the Decrease of Plasma Membrane and Intracellular Cholesterol Level

**Figure 4:**
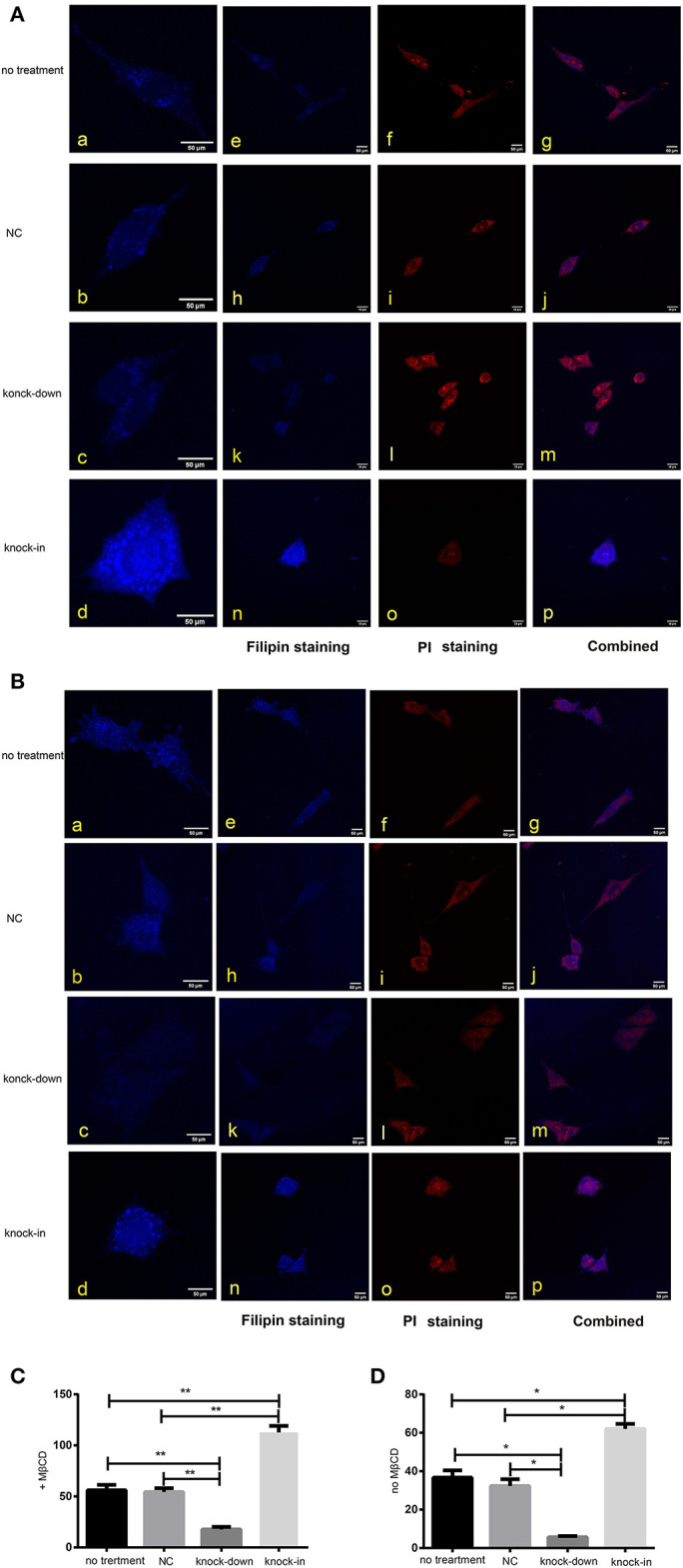
DHCR24 knock-down lead to the decrease of plasma membrane and intracellular cholesterol level. Whole-cell and intracellular cholesterol stained with filipin. **(A)** Filipin stained cholesterol in the intracellular compartments. Cells were treated with MβCD (methyl-β-cyclodextrin) before fixation and filipin staining. The cells in blue were stained with filipin, and in red were stained with PI (propidium iodide), the final column shows a merged image of the two channels. **(B)** Whole-cell cholesterol staining with filipin on fixed SH-SY5Y cells. Filipin stains cholesterol in the plasma membrane and in intracellular compartments. Scale bar = 50 μm. (a–d) of **(A,B)** were the zoomed picture of each group. Filipin intensity of cholesterol in the intracellular compartments **(C)** and cholesterol in whole-cell **(D)** were measured using Image J Software. All the data are presented as mean ± SD of at least three individual experiments (*n* = 3). *P < 0.05, **P < 0.01; vs. the no treatment and NC group.

As a major constituent of cellular membranes, cholesterol is essential in providing the membrane with its mechanical and structural properties, but also critical in the formation of membrane lipid-raft domains (Caveolae) (Matthews et al., 2008; Zerenturk et al., 2013). DHCR24 is known as a synthetase heavily involved in cholesterol synthesis, controlling the cholesterol synthesis in the brain (Greeve et al., 2000; Drzewińska et al., 2009). Filipin III is naturally fluorescent and specifically binds to free cholesterol, and it labels total cellular cholesterol, especially the plasma membrane. In addition, to visualize intracellular cholesterol pools, we treated live cells with methyl-β-cyclodextrin (MβCD), a molecule able to deplete cholesterol from the plasma membrane (Zidovetzki and Levitan, 2007). Intracellular and plasma membrane cholesterol were labeled and analyzed, respectively, by Filipin III staining. In our study, whole-cell and intracellular cholesterol were stained with filipin III and analyzed by a confocal laser scanning microscope. We found that fluorescence intensity of plasma membrane and intracellular cholesterol in the DHCR24 knock-down group was obviously weaker than that of no treatment group and vector group cells, suggesting the decrease of plasma membrane and intracellular cholesterol level (Figures 4A,B). In contrast, as shown in Figures 4C,D, immunofluorescence intensity analysis revealed that the level of plasma membrane and intracellular cholesterol were markedly increased in the DHCR24 knock-in group cells compared to the control group cells, suggesting the increase of plasma membrane and intracellular cholesterol level. Together, we verified that DHCR24 knock-down obviously lead to the decrease of plasma membrane cholesterol and intracellular cholesterol level.

### DHCR24 Knock-Down Lead to Inhibition of PI3-K/Akt Signaling in a Membrane Lipid Raft-Dependent Manner

**Figure 5:**
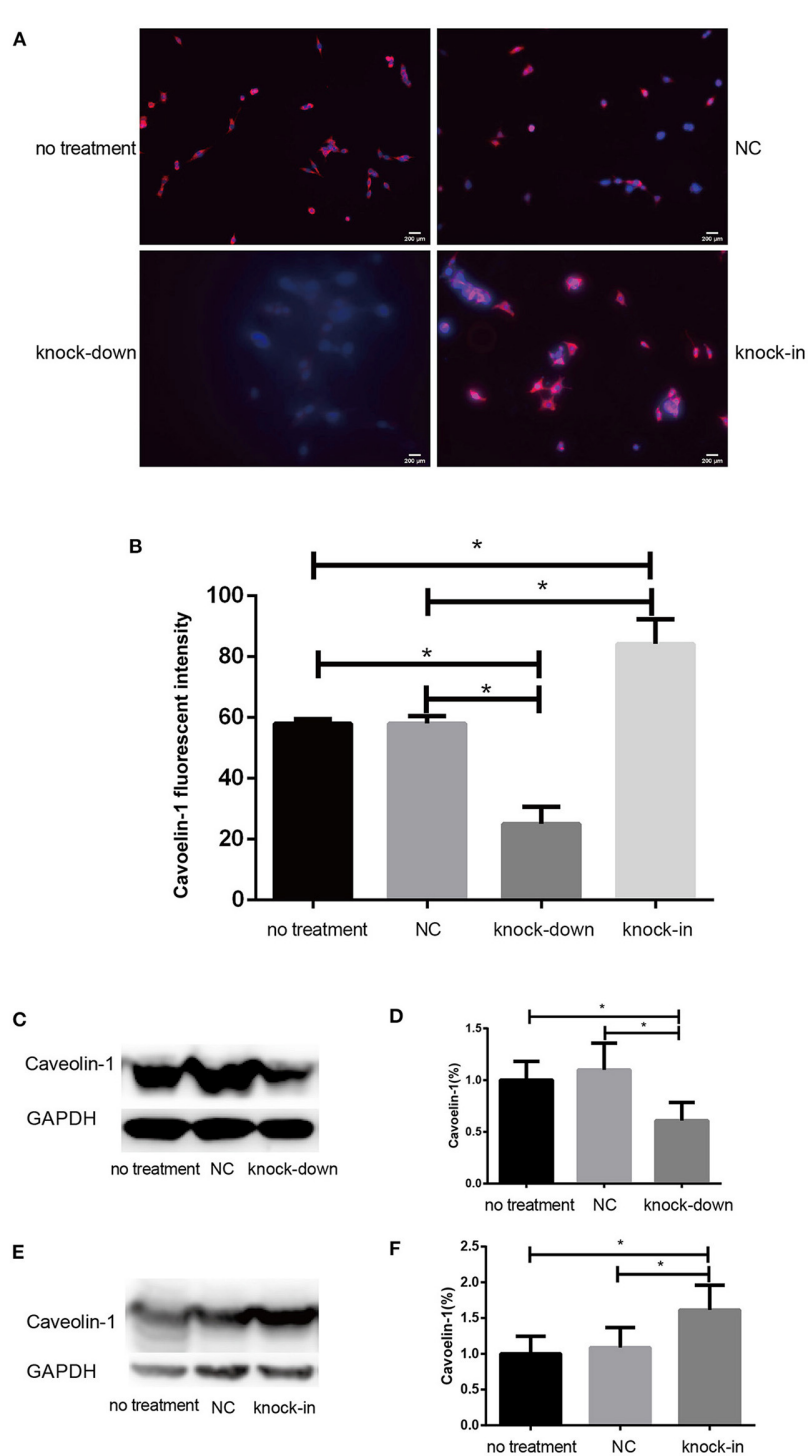
DHCR24 knock-down lead to downregulation of caveolin-1 in plasma membrane caveolae in SH-SY5Y cells. **(A)** Immunofluorescence studies showing staining of caveolin-1 in SH-SY5Y cells. Scale bar=200 μm. **(B)** The fluorescence intensities of caveolin-1 were quantified and presented as the mean of IOD of Caveolin-1 immunofluorescence intensity. **(C,D)** Western blots of caveolin-1 from DHCR24 knock-down SH-SY5Y cells. **(E,F)** Western blots of caveolin-1 from DHCR24 knock-in SH-SY5Y cells. All the data are presented as mean ± SD of at least three individual experiments (*n* = 3). *P < 0.05, **P < 0.01; vs. the no treatment and NC group. caveolin-1, in red-fluorescence; DAPI, in blue-fluorescence (Figures 2M, 5C share one loading control).

Consistent with our findings analyzed by Filipin III staining, some data suggest that the knock-down or deficiency of DHCR24 lowers cholesterol level of neuronal cell membrane and leads to cholesterol depletion in lipid rafts of cell membranes (Lu et al., 2006; Matthews et al., 2008; Gao et al., 2011). The cellular cholesterol biosynthesis is critical for the activation and maintenance of the PI3-K/Akt cell survival cascade (Lu et al., 2006; Gao et al., 2011). In this study, we examined a possible role of caveolin-1 related lipid raft in the PI3-K/Akt signal transduction in SH-SY5Y cells. In present study, western blot analysis showed that the expression of caveolin-1 protein was markedly lowered in DHCR24 knock-down SH-SY5Y cells compared to control groups, and concomitantly its fluorescence intensity of cell caveolin-1 in DHCR24 knock-down group was weaker than that of control group cells by immunofluorescence analysis, suggesting the disruption of membrane lipid-raft/caveolae in SH-SY5Y cells (Figures 5A–D). Conversely, as shown in DHCR24 knock-in group, immunofluorescence and western blot analysis revealed that DHCR24 knock-in significantly upregulated the expression of caveolin-1 protein in the caveolae fraction of the SH-SY5Y cells, suggesting the enhancement of caveolae in SH-SY5Y cells (Figures 5A,B,E,F). Based on our findings, we confirmed that DHCR24 knock-down could simultaneously decrease of plasma membrane cholesterol and caveolin-1 in the lipid-raft/caveolae. According to previous data and our study, we demonstrated that DHCR24 knock-down impaired the plasma membrane lipid raft structure and function which is mediated by the depletion of cholesterol and caveolin-1.

**Figure 6:**
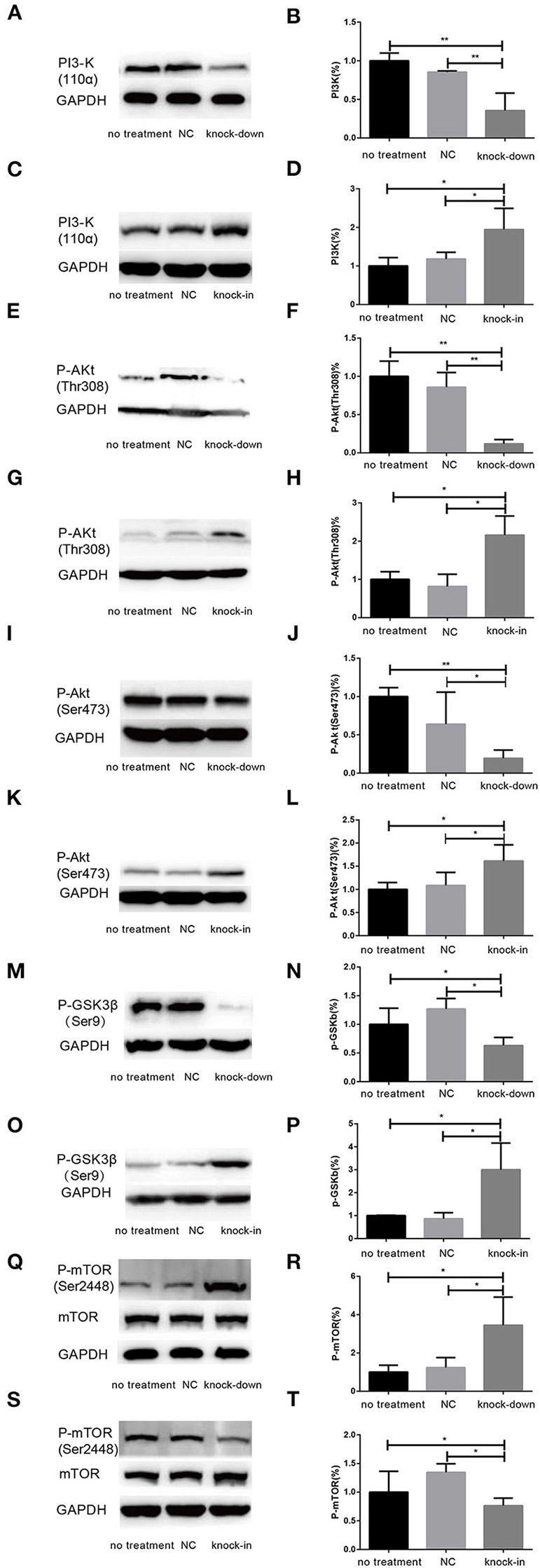
DHCR24 knock-down induced the overactivation of GSK3β/mTOR signaling via PI3K/Akt pathway. **(A,B,E,F,I,J,M,N,Q,R)** Representative western blots of total PI3-K, p-Akt at Ser473, p-Akt at Thr308, p-GSK3β at Ser9, total mTOR, p-mTOR at Ser2448 from DHCR24 knock-down SH-SY5Y cells. **(C,D,G,H,K,L,O,P,S,T)** Representative western blots of total PI3-K, p-Akt at Ser473, p-Akt at Thr308, p-GSK3β at Ser9, total mTOR, p-mTOR at Ser2448 from DHCR24 knock-in SH-SY5Y cells. All the data are presented as mean ± SD of at least three individual experiments (*n* = 3). *P < 0.05, **P < 0.01; vs. the no treatment and NC group (Figures 1C, 2E, 6A,Q share one loading control).

Additionally, Cholesterol and caveolin-1 are essential components of caveolae (Chini and Parenti, 2004). Caveolae, cholesterol-rich lipid-raft microdomains of plasma membrane, has been known to modulate the function of lipid raft-dependent protein kinases, such as PI3-K kinase (Chini and Parenti, 2004; Lu et al., 2006; Matthews et al., 2008; Gao et al., 2011). Here, we further evaluate the activation of PI3-K and Akt induced by the knock-down or knock-in of DHCR24 in SH-SY5Y cells. In our experiments, we found that level of total Akt was similar among the four groups (data not shown, see Supplementary Data). As shown in Figure 6, compared with the no treatment and negative control group, the level of total I class PI3-K (p110α), Phospho-Akt (Thr308) and Phospho-Akt (Ser473) were significantly decreased by DHCR24 knock-down in SH-SY5Y cells, leading to the inhibition of Akt kinase (Figures 6A,B,E,F,I,J). Conversely, as shown in Figure 6, the level of total PI3-K, p-Akt (Thr308) and p-Akt (Ser473) were obviously increased in DHCR24-in SH-SY5Y cells, suggesting the activation of Akt kinase, which is similar to the previous study (Lu et al., 2006). To sum up, above data further demonstrated that DHCR24 knock-down impaired the lipid raft organization of caveolae and thereby inhibited the activation of PI3-K/Akt survival signaling via lipid raft dependent manner.

### DHCR24 Knock-Down Induced the Overactivation of GSK3β/mTOR Signaling via PI3K/Akt Signaling

As a downstream substrate of PI3-K/Akt Signaling, GSK3β is a Ser/Thr kinase that is active in its non-phosphorylated form, and negatively regulated its activity mainly by Akt kinase (Buller et al., 2008; Bhaskar et al., 2009). So, quantitative analysis of the total GSK3β and phospho-GSK3β (Ser9) was also detected by western blot in different groups. In the study, by DHCR24 knock-down in SH-SY5Y cells, western blot analysis indicated that the level of total GSK3β was similar among the different groups (data not shown, see Supplementary Data). In contrast, compared with the control groups, the level of GSK3β phosphorylated at Ser9 was significantly lowered in the DHCR24 knock-down group (Figures 6M,N). Conversely, compared with the control group, the level of Phospho-GSK3β (Ser9) was markedly increased in DHCR24 knock-in group (Figures 6O,P). GSK3β is inactive when phosphorylated at Ser9; and the level of GSK3β phosphorylated at Ser9 inversely correlate with its activity (Buller et al., 2008; Bhaskar et al., 2009). Concomitantly, with the inhibition of PI3-K/Akt signaling (Figures 6A,B,E,F,I,J), DHCR24 knock-down decrease the level of p-GSK3β (Ser9), strongly suggesting the activation of GSK3β kinase in SH-SY5Y cells. In summary, our outcomes suggested that DHCR24 knock-down obviously increase the activity of GSK3β signaling through PI3-K/Akt signaling.

Abnormal activation of mTOR signaling have been recently implicated in the pathophysiology of AD and other neurodegenerative disease, including such as autophagy, tauopathy, and Aβ metabolism (Buller et al., 2008; Bhaskar et al., 2009; Zare-Shahabadi et al., 2015). However, in DHCR24 knock-down SH-SY5Y cells the implication of mTOR signaling still remains unknown. Further, we found that DHCR24 knock-down significantly and simultaneously decreased the level of phosphorylated GSK3β at Ser9 (inactive form) and increased the level of phosphorylated mTOR at Ser2448 (active form), and concomitantly phosphorylated Akt at Ser308/Ser473 were also significantly decreased in SH-SY5Y cells when compared with control cells, as shown in Figures 6E,F,I,J,M,N,Q,R. On the contrary, as shown in Figure 6, DHCR24 knock-in significantly increased the level of phosphorylated Akt at Ser308/Ser473 and increased the level of phosphorylated GSK3β at Ser9, and concomitantly decreased the level of phosphorylated mTOR at Ser2448 (Figures 6G,H,K,L,O,P,S,T). Thus, our results showed the activation of mTOR signaling in knock-down DHCR24 SH-SY5Y cells in comparison to control cells. However, mTOR integrates multiple signaling pathways, such as upstream components Akt and GSK3β. As a major downstream target of the PI3-K/Akt/mTOR pathway, the Akt activation usually lead to the increase of p-mTOR (Ser2448), and conversely the Akt inactivation can induce the decrease of p-mTOR (Ser2448) (Buller et al., 2008; Bhaskar et al., 2009). Moreover, GSK3β activation can also promote phosphorylated activation of mTOR (Ser2448) (Buller et al., 2008; Bhaskar et al., 2009). So, our data supported the activation of mTOR signaling could result from the stimulation of GSK3β signaling, and do not depend on the stimulation of PI3K/Akt signaling. Therefore, our results suggested that the knock-down of DHCR24 promoted the activation of mTOR signaling via PI3-K/Akt/GSK3β.

## Discussion

In present study, in SH-SY5Y cells, we found that silencing DHCR24 facilitated abnormal hyperphosphorylation of the tau sites, including Thr181, Ser199, Thr231, Ser262, and Ser 396. Conversely, the DHCR24 knock-in could abolish hyperphosphorylation of these p-tau sites. Very importantly, our results are tightly consistent with previous studies showing that Thr181, Ser199, Thr231, Ser262, and Ser 396 are major phosphorylation sites of p-tau in the progression of AD (Duka et al., 2013; Wang et al., 2013; Neddens et al., 2018). In Alzheimer disease, tau was hyperphosphorylated at over 80 epitopes of p-Tau (Wang et al., 2013). The accumulating data supported that the altered phosphorylation of some tau sites, such as Tau site at Thr181, Ser199, Thr231, Ser262, and Ser422, can lead to generate a gain of toxic function for tau (Wang et al., 2013; Neddens et al., 2018). P-tau could self-assemble into soluble aggregates of oligomeric tau, paired helical filaments (PHF), straight filaments (SF), or neurofibrillary tangles (NFT), and also convert tau into a prion-like protein, which are critical events in the pathological progression of AD (Duka et al., 2013; Moszczynski et al., 2015; Alonso and Cohen, 2018). Furthermore, it appears that phosphorylation of tau at Thr231 and Ser262 is critically involved in their self-assembly into PHF because phosphorylation of Thr231 and Ser262 appears to be necessary for the conversion of normal tau to pathological tau (Ikura et al., 1998; Alonso and Cohen, 2018; Alonso et al., 2018). Collectively, the abnormal hyperphosphorylation of tau not only lead to the loss of its normal function but also the gain of a toxic activity. Based on previous data, we confirmed that the altered phosphorylation of these tau sites induced by DHCR24 knock-down might be a critical event. In a word, we found that the abnormal phosphorylation of several toxic tau sites was obviously induced by DHCR24 downregulation. Therefore, our findings for the first time unraveled a relationship between DHCR24 downregulation and tauopathy.

**Figure 7:**
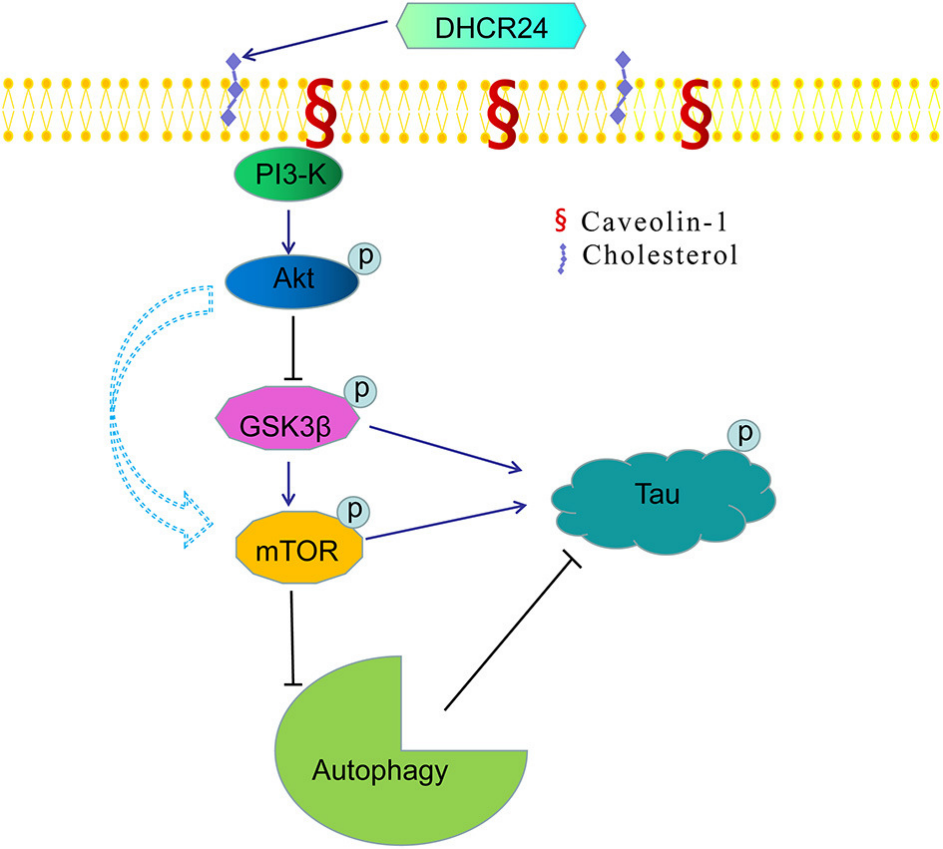
A bridge connecting cholesterol and AD: knock-down of DHCR24 mediated disregulation of cholesterol homeostasis in regulating tau hyperphosphorylation and autophagy. DHCR24 knock-down could lead to the abnormality of membrane lipid raft structure and function, then in turn resulting in the inhibition of lipid raft-dependent PI3-K/Akt signaling. Moreover, it also enhanced the activation of GSK3β and mTOR signaling by lipid raft-dependent PI3-K/Akt signaling. In addition, DHCR24 knock-down obviously induced tau hyperphosphorylation and inhibition of autophagy by the overactivation of GSK3β and mTOR signaling via lipid raft-dependent PI3-K/Akt signaling, which are involved in the pathogenesis of AD.

Noticeably, in our study, *immunofluorescence and western blot analysis revealed* that the cholesterol level of plasma membrane and the expression level of membrane lipid raft-anchored caveolin-1, the principal structural protein of caveolae, were markedly lowered in SH-SY5Y cells with silencing DHCR24. Conversely, DHCR24 knock-in significantly increased the level of plasma membrane cholesterol and lowered the expression of membrane caveolin-1. Concomitantly, we found that the level of total class I PI3-K, Phospho-Akt (Ser473) and Phospho-Akt (Thr308) were also markedly lowered by DHCR24 knock-down in SH-SY5Y cells. In contrast, the DHCR24 knock-in obviously increased the expression level of total class I PI3-K and the Phospho-Akt (Ser473 and 308), leading to the enhancement of PI3-K/Akt signaling. Thus, we considered that the decrease of PI3-K expression induced by DHCR24 knock-down could directly inhibited the phosphorylation activation of Akt, leading to the inhibition of PI3-K/Akt signaling. In addition, previous study suggested that brain cholesterol deficiency induced by silencing DHCR24 was associated with reduce of membrane cholesterol and altered membrane cholesterol-rich lipid-raft composition including the dysfunction of caveolae in cholesterol-rich lipid-raft microdomains, resulting in the markedly Akt inactivation, which is partly consistent with our outcomes (Lu et al., 2006). And cholesterol-rich caveolae in membrane lipid-rafts control a great variety of biological functions through their regulation of enzymes, receptors, neurotransmitter (Lynch and Mobley, 2000; Chini and Parenti, 2004). Moreover, it has been demonstrated that the main activation of Akt is done by PDK1 at cellular membranes and initiated by lipid raft-dependent kinases of the class I PI3K family (Lynch and Mobley, 2000; Chini and Parenti, 2004; Gao et al., 2011). To sum up, as the decline of cholesterol biosynthesis and caveolin-1 expression induced by DHCR24 knock-down, the disruption of lipid rafts*/caveolae* may lead to the alteration of raft-dependent functional protein kinase, such as PI3-K kinase (Figure 7). Together, DHCR24 knock-down lead to the decrease of cholesterol synthesis, the reduction of plasma membrane cholesterol and alteration of membrane lipid raft structure and function, and then induced the inhibition of PI3-K/Akt signaling.

In present paper, we also further showed that the DHCR24 knock-down could induce overactivation of GSK3β and mTOR kinase activity by the inhibition of PI3-K/Akt. Conversely, the DHCR24 knock-in could promote the activation of PI3-K/Akt signaling, and simultaneously inhibition of GSK3β and mTOR signaling. Furthermore, GSK3β was one of the first identified substrates of the heavily studied oncogenic kinase Akt, phosphorylation by which inhibits GSK3β activity. Akt activation is highly dependent on the class I PI3-K, and in turn promte the GSK3β at Ser9 phosphorylation and inhibition of GSK3β activity (Buller et al., 2008; Bhaskar et al., 2009). Obviously, in present study, overactivation of GSK3β signaling could be dependent on the inhibition of PI3-K/Akt. Thus, we firstly confirmed that DHCR24 knock-down induced the overactivation of GSK3β signaling through PI3-K/Akt pathway. Besides, it has been confirmed that mTOR activation is controlled by a variety of upstream components, including PI3-K/Akt and GSK3β (Bhaskar et al., 2009). In PI3-K/Akt/mTOR pathway manner, active p-Akt promotes the activation of mTOR through phosphorylation of the tuberous sclerosis complex 2 (TSC2), and active GSK3β has also been shown to phosphorylate mTOR and induce the activation of mTOR (Buller et al., 2008; Bhaskar et al., 2009). In present experiment, with the inhibition of PI3-K/Akt signaling by DHCR24 knock-down, leading to the inhibition of Akt signaling and the enhancement of GSK3β signaling, so the activation of mTOR signaling could be activated by the enhancement of GSK3β signaling through the inhibition of PI3-K/Akt pathway, but could be not directly dependent on inactive p-Akt. To sum up, mechanistically, the overactivation of mTOR was mediated by GSK3β through the inhibition of PI3-K/Akt pathway. Collectively, we showed that DHCR24 knock-down could induce the enhancement of GSK3β/mTOR signaling axis by PI3-K/Akt pathway.

In addition, the alteration of GSK3β and mTOR signaling plays a crucial role in tauopathy, which directly or indirectly participated in regulation of tau phosphorylation (Balaraman et al., 2006; Buller et al., 2008). In our study, we found that the silencing DHCR24 could induce the inhibition of PI3-K/Akt, simultaneously leading to the overactivation of GSK3β and mTOR kinase activity, as well as concomitantly the hyperphosphrylation of tau at Thr181, Ser199, Thr231, Ser262, and Ser 396 in SH-SY5Y cells (Figures 2, 6). On the contrary, the DHCR24 knock-in could simultaneously reversed the effects of silencing DHCR24 on the inhibition of PI3-K/Akt signaling, overactivation of GSK3β and mTOR signaling, and the hyperphosphrylation of tau at Thr181, Ser199, Thr231, Ser262, and Ser 396 in SH-SY5Y cells (Figures 2, 6). Previous data suggest that the GSK3β is one of main tau kinases, which nearly could phosphorylate tau at almost all the sites (Balaraman et al., 2006; Alejandra et al., 2010; Hernandez et al., 2013; Llorens-Marítin et al., 2014). Moreover, GSK3β participate in the phosphorylation of tau at multiple sites, which appear to be required to convert it into the pathological protein. These sites include four clusters amino terminal to the microtubule binding domains, i.e. Thr181, Ser199/Thr205, Thr212/Thr217, Thr231/Ser235, Ser262, and Ser396/Ser422 regions (Alejandra et al., 2010; Llorens-Marítin et al., 2014; Alonso and Cohen, 2018; Alonso et al., 2018). Additionally, mTOR, another tau kinase, is involved in the regulation of tau phosphorylation. However, the mechanism of tauopathy mediated by mTOR is still unclear. Firstly, evidences suggest that activation of mTOR induces p-tau production and aggregation by GSK3β, and autophagy by mTOR (Caccamo et al., 2010; Yang and Klionsky, 2010; Zare-Shahabadi et al., 2015). Secondly, it has been reported that the activation of mTOR enhanced the expression levels of p70S6K and p4E-BP1 which further lead to the tauopathy (Caccamo et al., 2010; Cai et al., 2015). Additionally, the hyperphosphorylated tau is found to be attenuated by rapamycin, a potential inhibitor of mTOR (Caccamo et al., 2010; Spilman et al., 2010; Cai et al., 2015). Thus, we firstly confirmed that DHCR24 knock-down lead to the hyperphosphorylation of tau protein, at least partly, by the enhancement of GSK3β/mTOR signaling axis through PI3-K/Akt pathway.

Dysfunction of autophagy mechanism has been proposed to play a critical role in AD and other neurodegenerative disorders (Zare-Shahabadi et al., 2015; Zhang et al., 2017; Chang et al., 2018). Moreover, autophagy-lysosome defects occur early in the pathogenesis of AD and have been proposed to be a significant contributor to the disease process (Zare-Shahabadi et al., 2015; Zhang et al., 2017). Here, we have demonstrated that DHCR24 knock-down simultaneously increased the expression of P62 protein and lowered LC3II/LC3I ratio, leading to autophagy suppression in SH-SY5Y cells. Concomitantly, DHCR24 knock-down also increased the overactivation GSK3β/mTOR signaling (Figures 3, 6). On the contrary, DHCR24 knock-in markedly promoted autophagy activity, and simultaneously lead to the inhibition of GSK3β/mTOR signaling in SH-SY5Y cells. LC3 and p62 are main markers for autophagy activity. P62/SQSTM1 is a multidomain protein that interacts with the autophagy machinery as a key adaptor of target cargo. P62 can directly interact with LC3 for autophagosome formation (Zhang et al., 2017). Further, the conversion of LC3-I to LC3-II denotes autophagy stimulation and autophagosome formation (Zare-Shahabadi et al., 2015). In addition, in our study, transmission electron microscope analysis revealed that the DHCR24 knock-down decreased autophagosome numbers in silencing SH-SY5Y cells compared to the blank or vector groups, suggesting the inhibition of autophagy (Figure 3). In contrast, DHCR24 knock-in reversed the effect of DHCR24 knock-down the decrease of autophagosomes. Furthermore, the autopagy-mediated dysfunction of tau protein and amyloid-β protein degradation mechanisms has been proposed to play a pivotal role in AD (Yang and Klionsky, 2010; Lee et al., 2013; Cai et al., 2015). Accordingly, with the tau hyperphosphorylation induced by DHCR24 knock-down in SH-SY5Y cells, the inhibition of autophagy could further cause the disturbance of homeostasis in p-tau clearance and deposition, involving in pathological process of tauopathy. Therefore, our outcomes strongly indicated that the DHCR24 knock-down obviously lead to the inhibition of autophagy through GSK3β/mTOR signaling axis.

Noteworthily, Ledesma et al. revealed that the neuronal membrane cholesterol have a significant reduction, the loss of neuronal membrane cholesterol and anomalous raft microdomains, which contribute to excessive amyloidogenesis in AD patients (Abad-Rodriguez et al., 2004; Ledesma et al., 2012). Besides, some data suggest there is a reduction in cholesterol in brain in aging and AD mice (Jacob et al., 2009; Ledesma et al., 2012; Llorens-Marítin et al., 2014; Mauricio et al., 2014). Generally speaking, in adult brain, differentiated neurons gradually lose their *de novo* synthetic ability and mainly rely on lipoprotein-conjugated cholesterol produced by Astrocytes (Dietschy and Turley, 2004). Thus, in the adult CNS cholesterol is mainly synthesized and regulated by astrocyte. The astrocyte meets neuronal cholesterol demands by secreting cholesterol-apolipoprotein complexes (Dietschy and Turley, 2004). Furthermore, some studies support that cholesterol synthetic genes in astrocytes were obviously down regulated in aging, diabetes and AD mice brain, suggesting the disruption of brain cholesterol biosynthesis and reduction in cholesterol synthesis in brain (Kleinridders et al., 2014; Orre et al., 2014; Lana et al., 2016; Boisvert et al., 2018; Han et al., 2020). It appears that there is an overall inhibition of cholesterol synthesis in astrocytes in neurodegenerative disorders, leading to the decrease of cholesterol synthesis and loss of cholesterol in brain. Very interestingly, in mice with knockout of SREBP2 in astrocytes, the loss of astrocyte cholesterol synthesis disrupted neuronal function and cognitive defects (Heather et al., 2017). Conversely, another important study showed that the loss of cholesterol in the hippocampus of aged mice produced a strong cognitive deficit, and cholesterol replenishment improved hippocampal cholesterol-loss-dependent cognitive decline (Mauricio et al., 2014). Similarly, DHCR24-deficient mouse brains had reduced levels of membrane cholesterol, and increased APP β-cleavage and Aβ production by disorganized cholesterol-rich lipid rafts, which showed similar reduction of membrane cholesterol than that reported in the AD patients (Abad-Rodriguez et al., 2004; Crameri et al., 2006; Ledesma et al., 2012). Based on our study and previous data, we propose that the decrease of plasma membrane cholesterol might tightly correlates with tauopathy and amyloid-beta pathology in AD and other neurodegenerative disorders.

In conclusion, in present study, our results further showed that DHCR24 could control the rate of cholesterol synthesis and cholesterol homeostasis by the Bloch pathway and/or K-R pathway. As a synthetase heavily involved in cholesterol synthesis, DHCR24 knock-down significantly lead to a reduction of cholesterol in plasma membrane and intracellular compartments, resulting in the cholesterol loss in SH-SY5Y cells. Furthermore, the decrease of plasma membrane cholesterol mediated by DHCR24 deficiency might contribute to the tauopathy in AD and other tauopathies, at least partly by membrane lipid raft-mediated signaling mechanism (Figure 7). Taken together, our outcomes provided convincing evidence supporting the importance of DHCR24 in the context of neurodegenerative disorders, highlighting the therapeutic potential and value in researching its activity further.

## Data Availability Statement

The raw data supporting the conclusions of this article will be made available by the authors, without undue reservation, to any qualified researcher.

## Author Contributions

HZ designed the study and wrote the manuscript. XB contributed to the experimental work about tau phosphorylation and autophagy. JW and MZ contributed to the transfection work of SH-SY5Y cells. YX, LD, and KY provided their experimental assistance and TEM work. JZ and JB contributed to analysis of data. YZ and GX provided their technical assistance. All authors have read the manuscript and provided the input.

## Conflict of Interest

The authors declare that the research was conducted in the absence of any commercial or financial relationships that could be construed as a potential conflict of interest.
