# Supplementary material for: DHCR24 Knock-Down Induced Tau Hyperphosphorylation at Thr181, Ser199, Thr231, Ser262, Ser396 Epitopes and Inhibition of Autophagy by Overactivation of GSK3β/mTOR Signaling
Source: Front Aging Neurosci. 2021 Apr 21;13:513605. doi: 10.3389/fnagi.2021.513605 (PMC8098657; doi:10.3389/fnagi.2021.513605)
Supplement: Supplementary file 1 [file Data_Sheet_1.pdf]

Supplementary Data

Figure 1. Supplementary Data - Figure 6. Supplementary Data

Figure 1. Supplementary Data

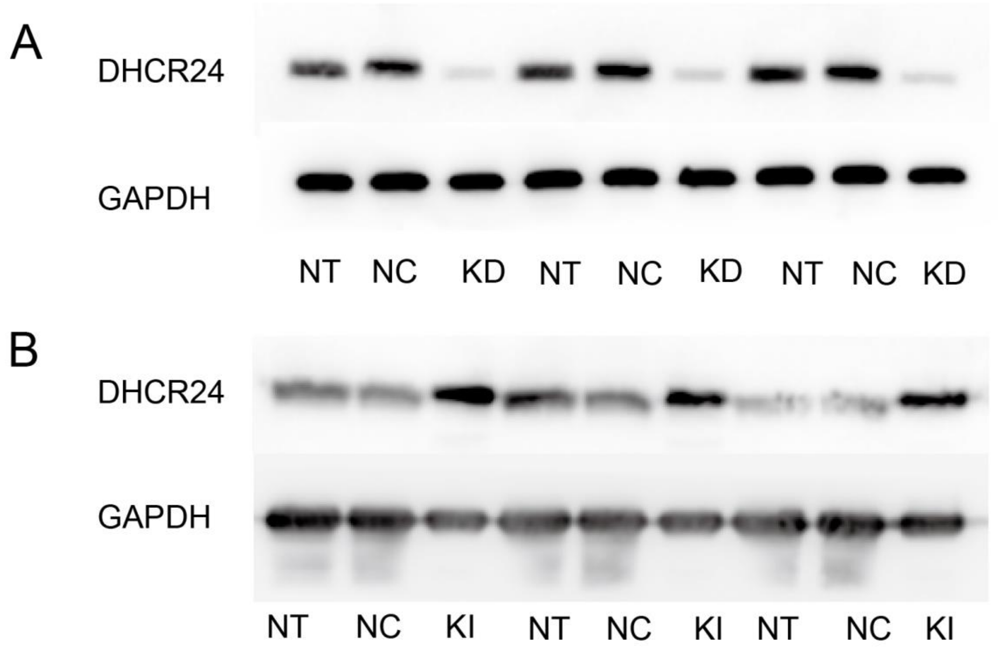

Supplementary data Figure 1 No treatment group was abbreviated with NT, negative control group was abbreviated with NC, knock-down group was abbreviated with KD, knock-in group was abbreviated with KI. Western blot was performed to analyze the knockdown (A) and knockin (B) of DHCR24.

Figure 2. Supplementary Data

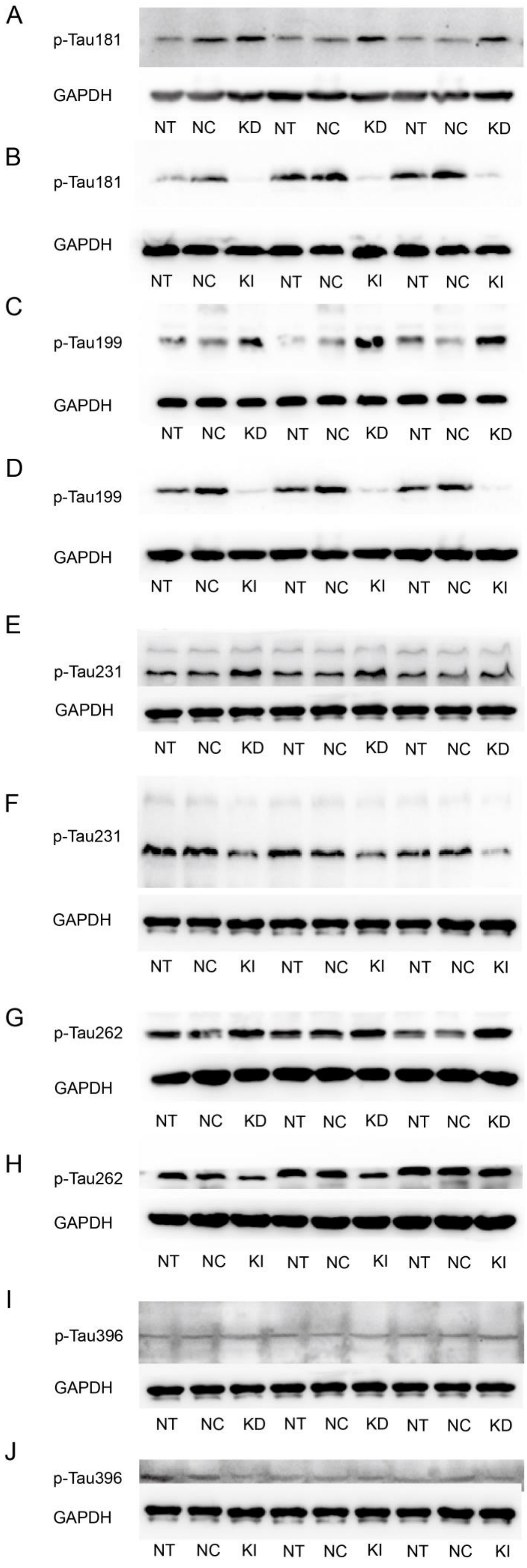

A
p-Tau181
GAPDH
NT NC KD NT NC KD NT NC KD
B
p-Tau181
GAPDH
NT NC KI NT NC KI NT NC KI
C
p-Tau199
GAPDH
NT NC KD NT NC KD NT NC KD
D
p-Tau199
GAPDH
NT NC KI NT NC KI NT NC KI
E
p-Tau231
GAPDH
NT NC KD NT NC KD NT NC KD
F
p-Tau231
GAPDH
NT NC KI NT NC KI NT NC KI
G
p-Tau262
GAPDH
NT NC KD NT NC KD NT NC KD
H
p-Tau262
GAPDH
NT NC KI NT NC KI NT NC KI
I
p-Tau396
GAPDH
NT NC KD NT NC KD NT NC KD
J
p-Tau396
GAPDH
NT NC KI NT NC KI NT NC KI

Supplementary data Figure 2 No treatment group was abbreviated with NT, negative control group was abbreviated with NC, knock-down group was abbreviated with KD, knock-in group was abbreviated with KI. (A, C, E, G, I) Representative western blots of phosphorylated tau at residues Thr181, Ser199, Thr231, Ser262 and Ser396 from DHCR24 knockdown SH-SY5Y cells. (B, D, F, H,J) Representative western blots of phosphorylated tau at residues Thr181, Ser199, Thr231, Ser262 and Ser396 from DHCR24 knockin SH-SY5Y cells.

Figure 3. Supplementary Data

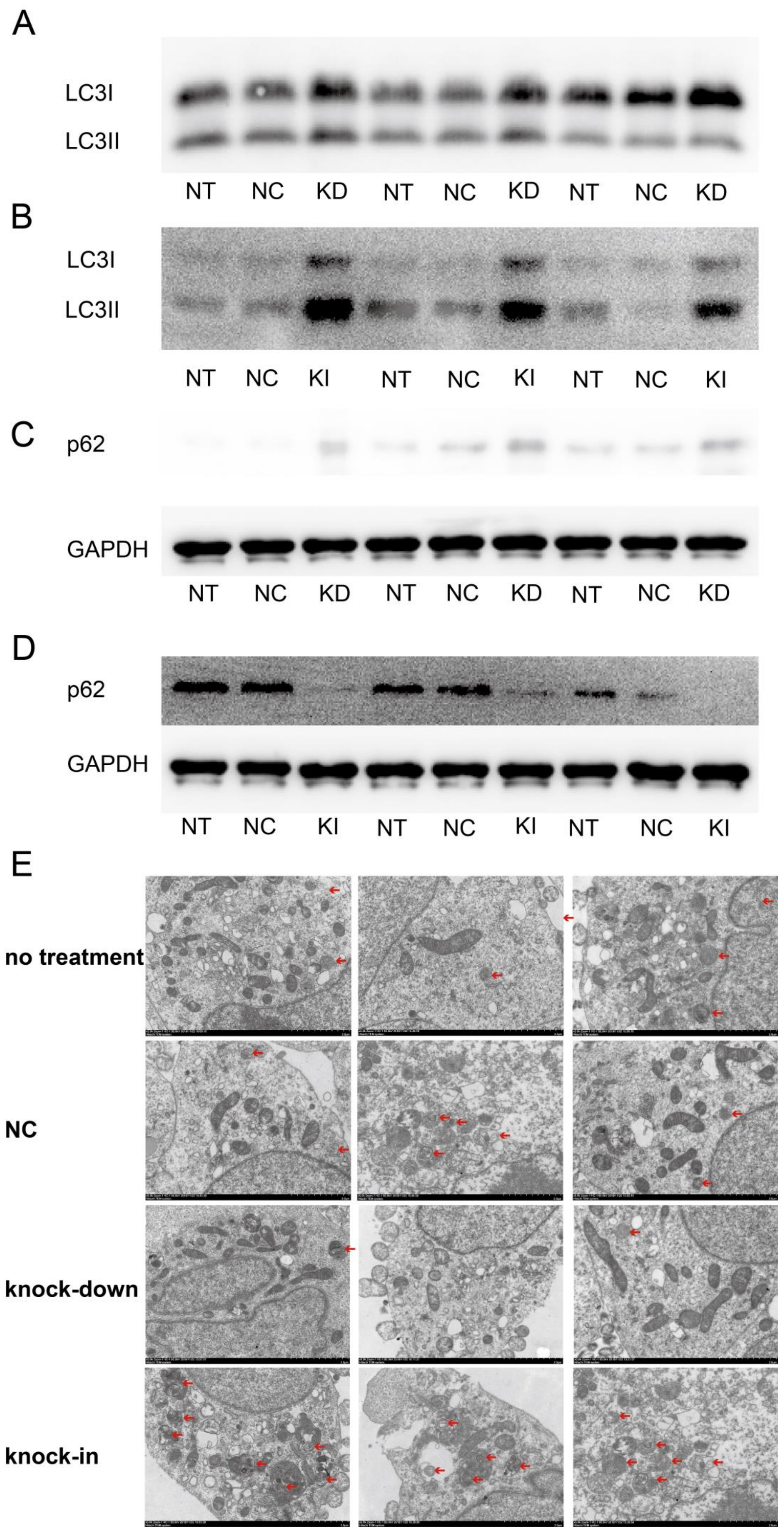
A
LC3I
LC3II
NT NC KD NT NC KD NT NC KD
B
LC3I
LC3II
NT NC KI NT NC KI NT NC KI
C
p62
GAPDH
NT NC KD NT NC KD NT NC KD
D
p62
GAPDH
NT NC KI NT NC KI NT NC KI
E
no treatment
NC
knock-down
knock-in

Supplementary data Figure 3 No treatment group was abbreviated with NT, negative control group was abbreviated with NC, knock-down group was abbreviated with KD, knock-in group was abbreviated with KI. (A, C) Representative western blots of LC3 and p62 from DHCR24 knockdown SH-SY5Y cells. (B, D) Representative western blots of LC3 and p62 from DHCR24 knockin SH-SY5Y cells. (E)Transmission electron microscopy showed the autophagosomes (red arrow). Scale bar = 2.0μm.

Figure 4. Supplementary Data

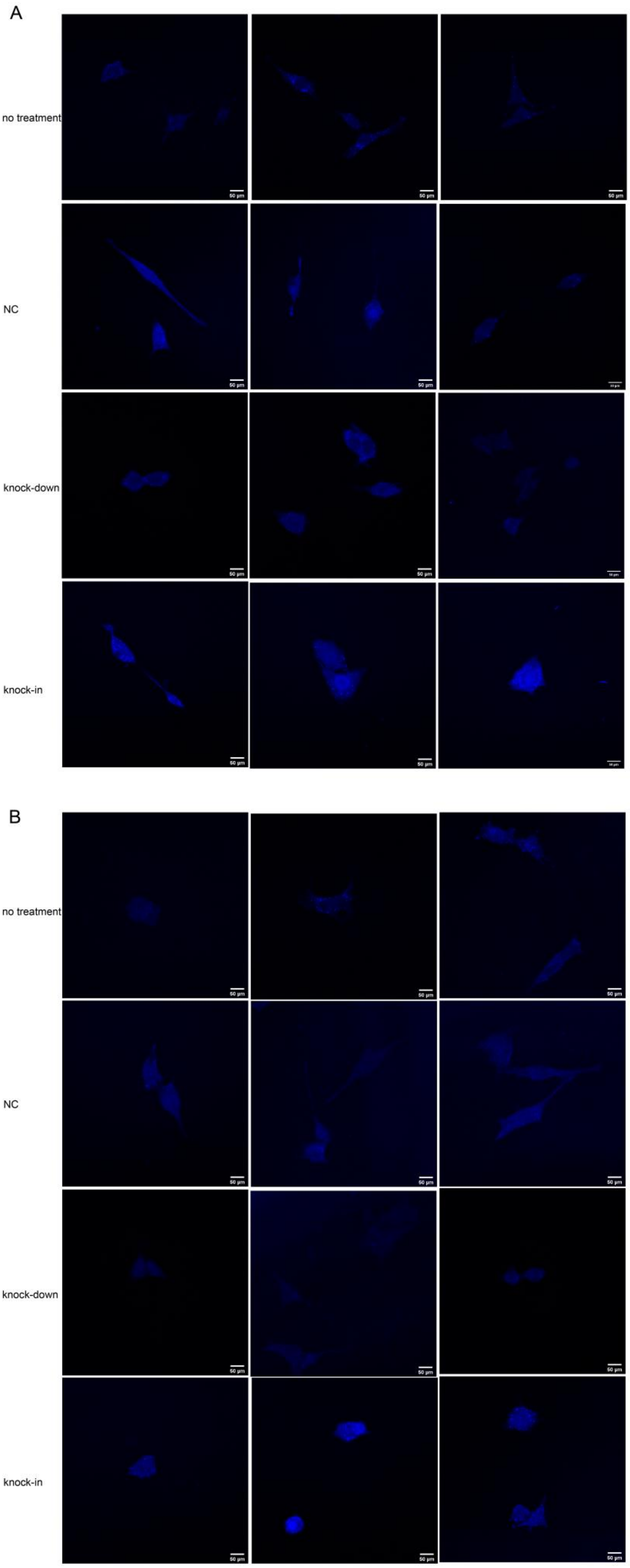
A
no treatment
NC
knock-down
knock-in
B
no treatment
NC
knock-down
knock-in

Supplementary data Figure 3 No treatment group was abbreviated with NT, negative control group was abbreviated with NC, knock-down group was abbreviated with KD, knock-in group was abbreviated with KI. (A) Filipin stained cholesterol in the intracellular compartments. (B) Filipin stained cholesterol in the whole-cell.

Figure 5. Supplementary Data

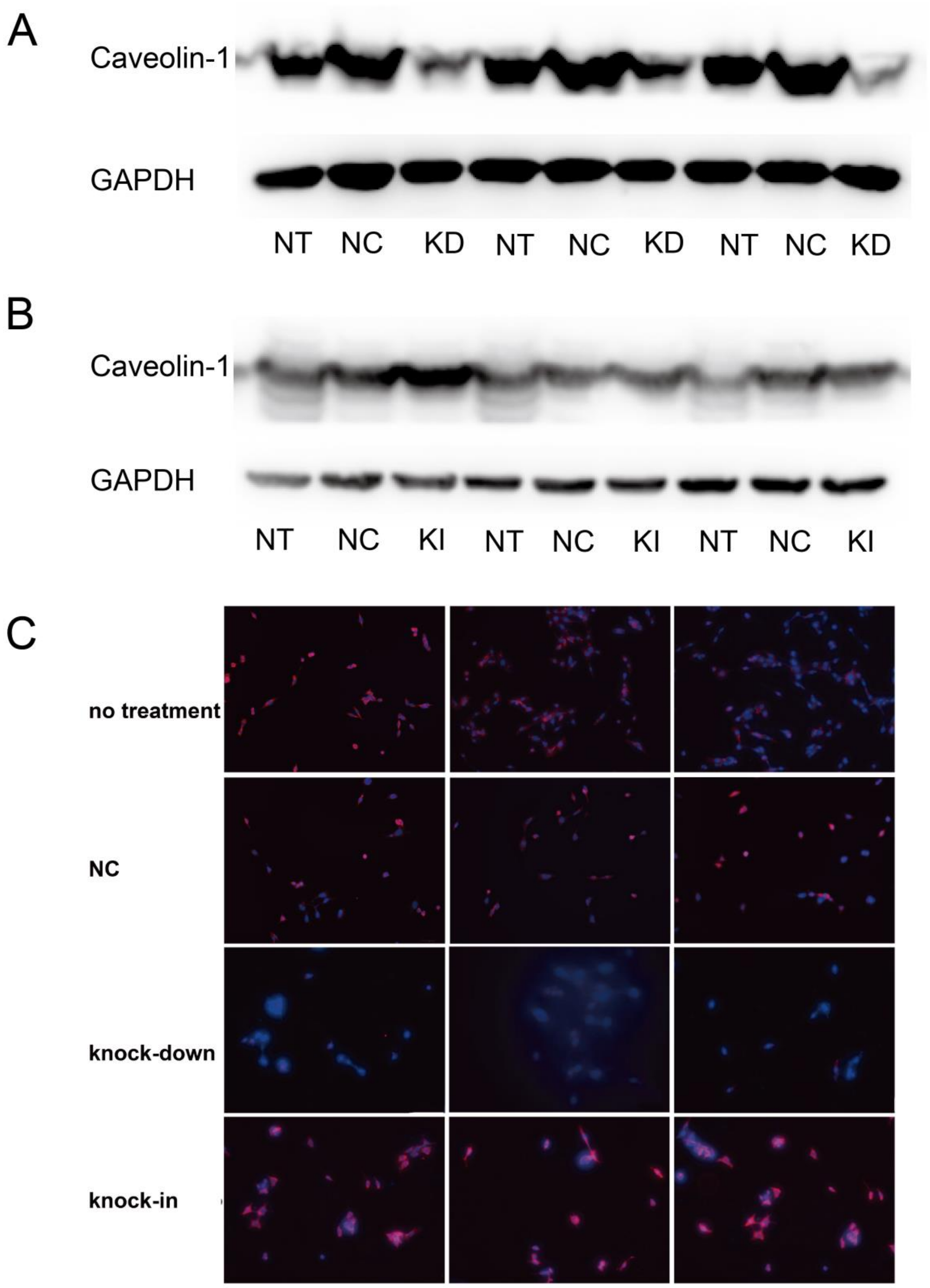

Supplementary data Figure 5 No treatment group was abbreviated with NT, negative control group was abbreviated with NC, knock-down group was abbreviated with KD, knock-in group was abbreviated with KI. (A) Western blots of caveolin-1 from DHCR24 knockdown SH-SY5Y cells. (B) Western blots of caveolin-1 from

DHCR24 knockin SH-SY5Y cells. (C) Immunofluorescence studies showing staining of caveolin-1 in SH-SY5Y cells.

Figure 6. Supplementary Data

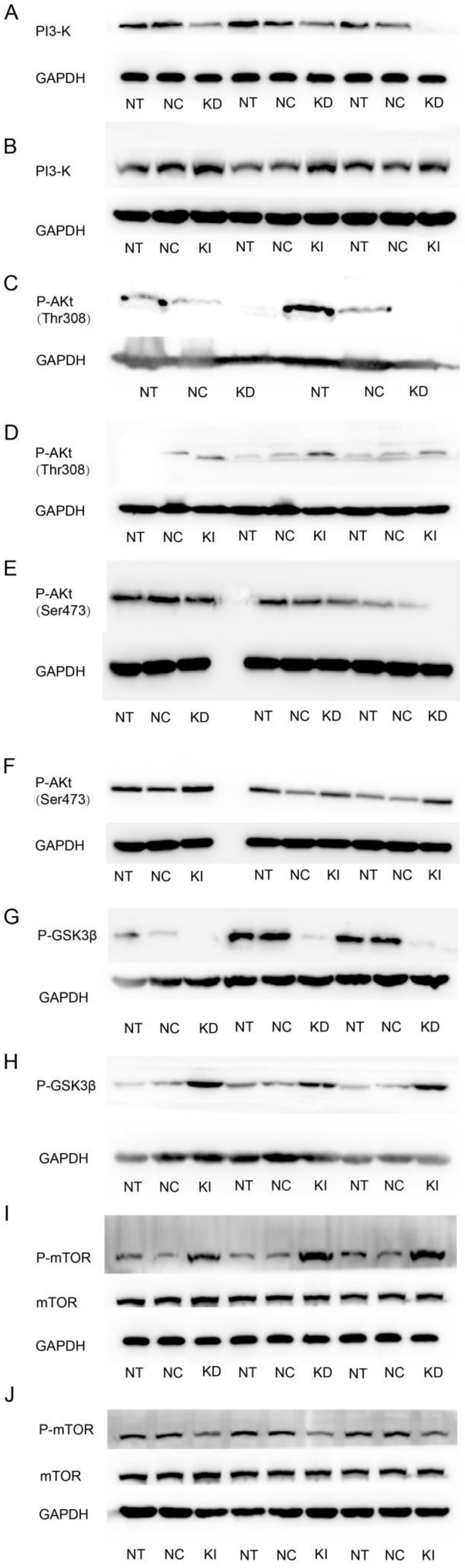
A
PI3-K
GAPDH
NT NC KD NT NC KD NT NC KD
B
PI3-K
GAPDH
NT NC KI NT NC KI NT NC KI
C
P-AKt (Thr308)
GAPDH
NT NC KD NT NC KD
D
P-AKt (Thr308)
GAPDH
NT NC KI NT NC KI NT NC KI
E
P-AKt (Ser473)
GAPDH
NT NC KD NT NC KD NT NC KD
F
P-AKt (Ser473)
GAPDH
NT NC KI NT NC KI NT NC KI
G
P-GSK3β
GAPDH
NT NC KD NT NC KD NT NC KD
H
P-GSK3β
GAPDH
NT NC KI NT NC KI NT NC KI
I
P-mTOR
mTOR
GAPDH
NT NC KD NT NC KD NT NC KD
J
P-mTOR
mTOR
GAPDH
NT NC KI NT NC KI NT NC KI

Supplementary data Figure 6 No treatment group was abbreviated with NT, negative control group was abbreviated with NC, knock-down group was abbreviated with KD, knock-in group was abbreviated with KI. (A, C, E, G, I) Representative western blots of total PI3-K, p-Akt at Ser473, p-Akt at Thr308, p-GSK3β at Ser9, total mTOR, p-mTOR at Ser2448 from DHCR24 knockdown SH-SY5Y cells. (B, D, F,H , J) Representative western blots of total PI3-K, p-Akt at Ser473, p-Akt at Thr308, p-GSK3β at Ser9, total mTOR, p-mTOR at Ser2448 from DHCR24 knockin SH-SY5Y cells.

Figure 7. Supplementary Data

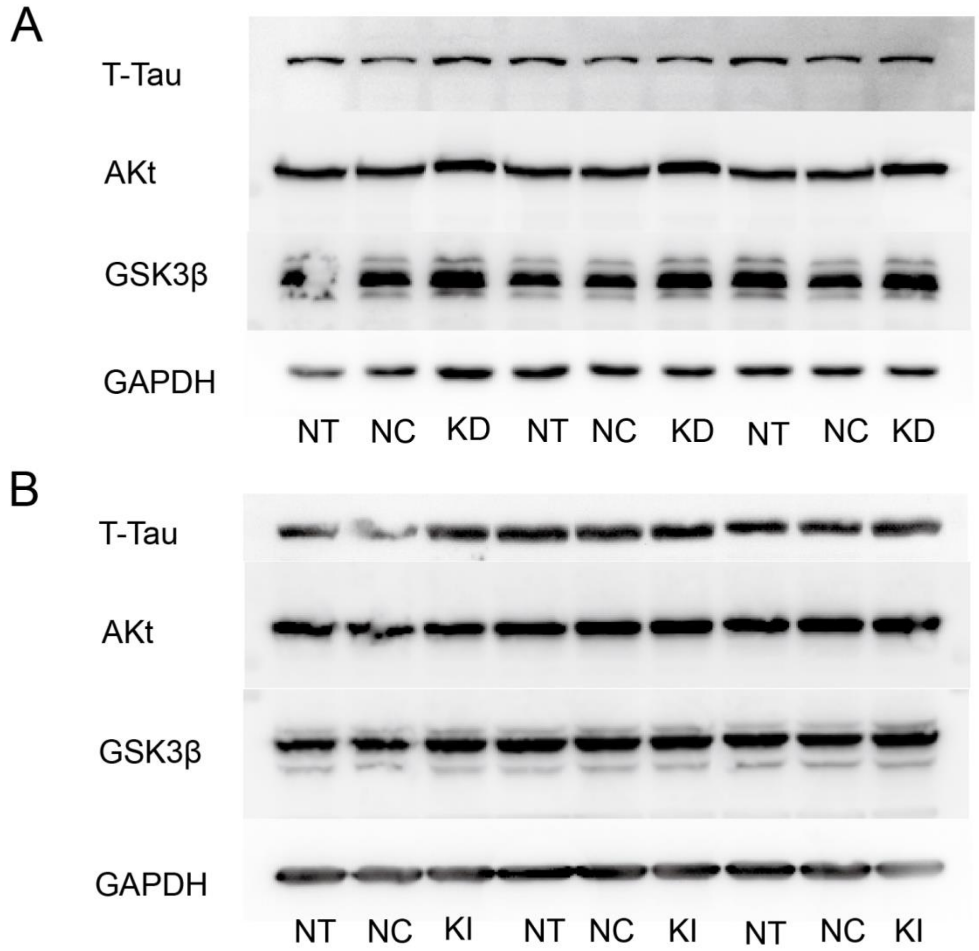

Supplementary data Figure 7 No treatment group was abbreviated with NT, negative control group was abbreviated with NC, knock-down group was abbreviated with KD, knock-in group was abbreviated with KI. (A) Representative western blots of total Tau, Akt and GSK3β from DHCR24 knockdown SH-SY5Y cells. (B) Representative western blots of total Tau, Akt and GSK3β from DHCR24 knockin SH-SY5Y cells.
